# Rapid Onset of Pulmonary Arterial Hypertension After Liver Transplant—A Case Report

**DOI:** 10.3390/reports9010083

**Published:** 2026-03-11

**Authors:** Simone Redaelli, Ryan Nazemian, Florian Hackl, Arun Uthayashankar, Michael Kaufman

**Affiliations:** Department of Anesthesiology, Perioperative, and Pain Medicine, Liver Transplant Anesthesia, Lahey Hospital & Medical Center, Burlington, MA 01805, USA

**Keywords:** pulmonary arterial hypertension, liver transplantation, *de novo* PAH, coronary artery disease, right heart, pulmonary hypertension

## Abstract

**Background and Clinical Significance**: Pulmonary hypertension (PH) is a recognized complication of chronic liver disease, most commonly manifesting as portopulmonary hypertension (POHP) prior to liver transplantation. While the natural history and management of pre-transplant PH are well described, the development of *de novo* pulmonary arterial hypertension (PAH) following liver transplantation remains exceedingly rare and poorly understood. In such cases, establishing true causality is challenging, and alternative explanations—including previously unrecognized or masked disease—must be carefully considered. This entity poses significant diagnostic and therapeutic challenges and may adversely affect post-transplant outcomes if not promptly recognized and treated. **Case Presentation**: We report the case of a 46-year-old man with end-stage liver disease secondary to alcohol use who underwent deceased donor liver transplantation without preoperative evidence of PH. His pre-transplant evaluation revealed preserved biventricular function and no measurable PH. Eight days postoperatively, he was readmitted with acute dyspnea, hypoxemia, and signs of right ventricular failure. Transthoracic echocardiography demonstrated severe right ventricular dilation and dysfunction with markedly elevated pulmonary artery systolic pressure. Right heart catheterization confirmed severe PAH. Secondary causes of PH were excluded. The patient was initiated on sildenafil and continuous intravenous epoprostenol, resulting in clinical, echocardiographic, and hemodynamic improvement. Subsequent follow-up demonstrated sustained response to therapy despite concurrent progression of coronary artery disease requiring complex percutaneous intervention. **Conclusions**: This case highlights a rare presentation of severe PAH occurring shortly after liver transplantation, in the absence of documented pre-transplant PH. While a causal relationship cannot be definitively established, the temporal association raises important clinical considerations. It underscores the need for heightened clinical vigilance for pulmonary vascular disease in post-transplant patients presenting with cardiopulmonary symptoms. Further research is warranted to elucidate the underlying mechanisms, risk factors, and optimal management strategies for PAH diagnosed after liver transplantation.

## 1. Introduction and Clinical Significance

Pulmonary hypertension (PH) is a known complication in chronic liver disease. It has been described in approximately 10 to 20% of patients with end-stage liver disease, with portopulmonary hypertension (POHP), a subtype of pulmonary arterial hypertension (PAH), being the most common cause (65%) of this disease [1,2]. POPH presents significant clinical challenges, particularly in the context of liver transplantation, where it has been associated with increased perioperative risk and poor outcomes if not appropriately managed. For these reasons, practice guidelines are available for POPH management in liver transplant candidates [3]. While the natural course of POPH and its implications for transplantation have been increasingly well-characterized, significantly less is known about the development of PH after liver transplantation. PAH development after liver transplantation remains exceedingly rare and its pathophysiology remains poorly understood. Importantly, it is often unclear whether such cases represent truly *de novo* disease or the unmasking of previously unrecognized pulmonary vascular pathology, particularly in the absence of pre-transplant overt clinical signs and symptoms [4,5].

Here, we present a case of rapid-onset, severe PAH following deceased donor liver transplant, occurring in a patient without prior overt evidence of PH.

This manuscript was prepared following the CARE guidelines (https://www.care-statement.org).

## 2. Case Presentation

**Figure 1:**
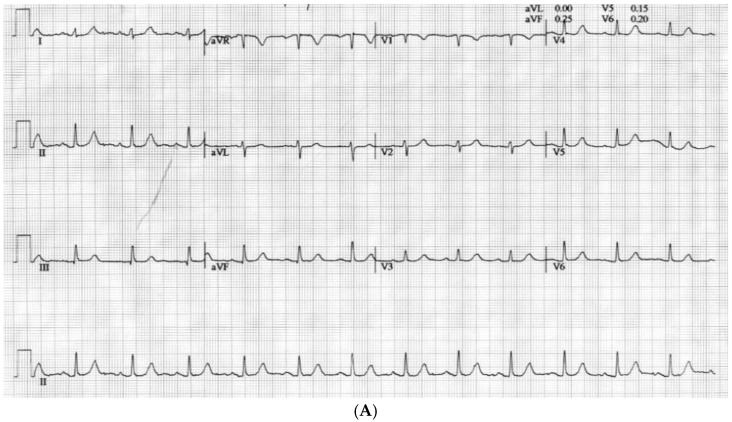

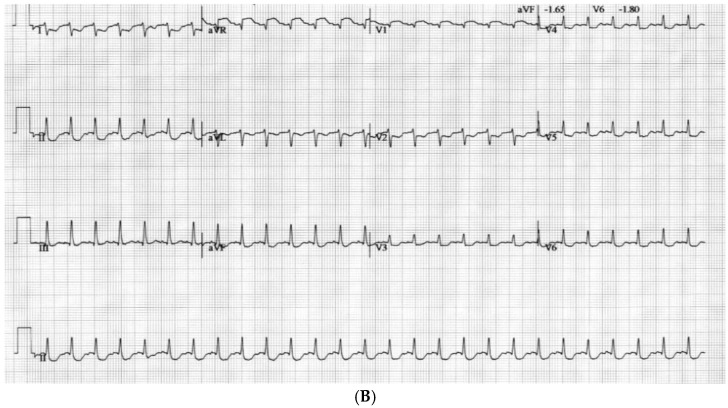
**Electrocardiogram performed during the** dobutamine stress test as part of the pre-transplant evaluation. Baseline (**A**) and peak stress (**B**) electrocardiogram. At baseline, the electrocardiogram appeared within normal limits, while at peak stress there were ST elevations in aVR and V1 along with diffuse ST depressions in other leads.

A 46-year-old male underwent a deceased donor liver transplant due to end-stage liver disease caused by alcohol. His past medical history included hypertension, dyslipidemia, class III obesity, gastroesophageal reflux disease, possible obstructive sleep apnea syndrome (OSAS), and smoking. During the pre-transplant evaluation, he reported exertional dyspnea and chest pressure at rest. The electrocardiogram at rest showed normal sinus rhythm. A preoperative echocardiogram was within acceptable limits (Video S1a,b). The pulmonary artery systolic pressure could not be estimated due to inadequate tricuspid regurgitation signal, although the right ventricle was normal in size and function. A saline contrast study showed late appearance of microbubbles in the left heart. A dobutamine stress echocardiogram indicated inducible ischemia in the anteroseptal region. Indeed, at peak stress, there were ST elevations in aVR and V1 along with diffuse ST depressions in other leads (Figure 1).

**Figure 2:**
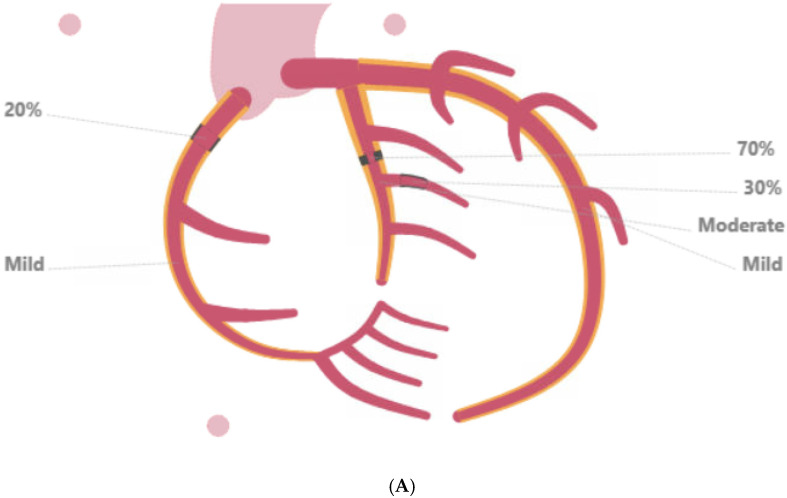

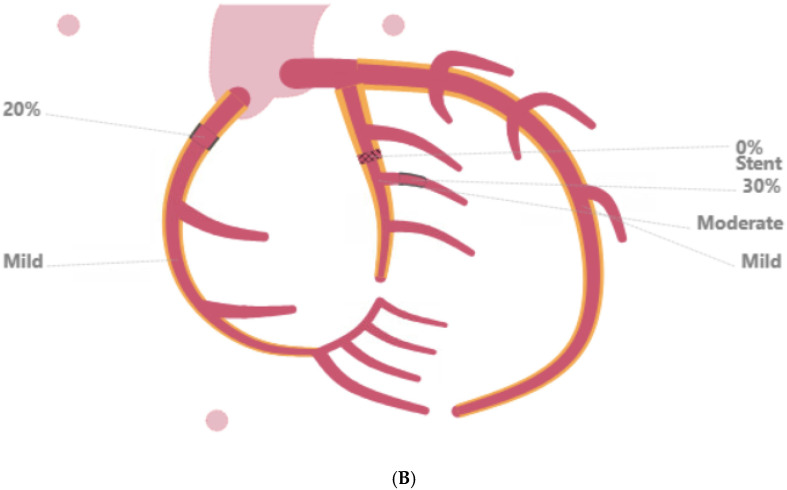
Panel (**A**) presents a diagnostic coronary angiography performed during pre-transplant evaluation. Mild diffuse disease in the left anterior descending coronary artery (LAD), moderate diffuse disease in the left circumflex (LCx) coronary artery with 70% stenosis in the mid-portion and 30% stenosis in the second obtuse marginal branch, and mild diffuse disease of the right coronary artery (RCA) with 20% stenosis of the proximal portion were found; panel (**B**) shows placement of a drug-eluting stent in the LCx with no residual stenosis.

Hypokinesis was observed in the mid to distal anterior, anteroseptum, and apical segments, and apex appeared hypokinetic at peak stress. Coronary arteries’ CT calcium score was 1066 with a distribution of 33 in the left main artery (LMA), 507 in the left anterior descending (LAD) coronary artery, 107 in the left circumflex coronary artery (LCx), and 419 in the right coronary artery (RCA) territory. Subsequent coronary angiography revealed mild diffuse disease throughout the LMA; moderate diffuse disease in the LCx, with 70% stenosis in the mid-portion and 30% stenosis in the second obtuse marginal branch; and mild diffuse disease of the RCA, with 20% stenosis of the proximal portion. A drug-eluting stent (DES) in the LCx was placed, followed by dual antiplatelet therapy (DAPT) which was discontinued after 2 months to ensure liver transplant listing (Figure 2).

From a pulmonology standpoint, the presence of obesity, along with daytime hypersomnolence, a history of witnessed apneas, and Epworth sleepiness scale of 12 raised suspicion of OSAS. Pulmonary function tests showed mild obstructive defect. At-home sleep study showed mild OSA, with apnea hypopnea index of 9/h and nadir SpO2 of 82%, and continuous positive airway pressure treatment was recommended, although it was not started before the transplant.

**Figure 3:**
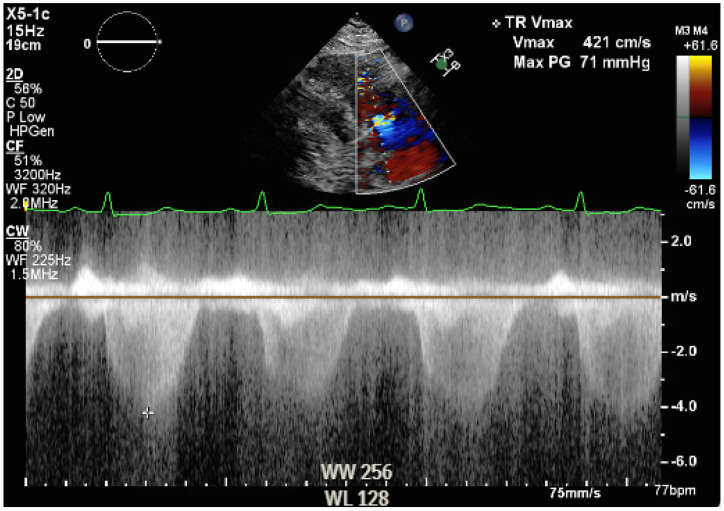
Pulmonary artery systolic pressure estimation.

The intraoperative course was not characterized by major complications. MELD 3.0 and MELD-Na were 24 and 23, respectively, on the day of surgery. The surgical procedure consisted of the piggyback technique and portal thrombectomy. Cold ischemic time was 5 h and 46 min, and warm ischemic time was 19 min. Intraoperatively, the patient’s hemodynamic parameters were stable and supported with phenylephrine and norepinephrine during the reperfusion and neo-hepatic phase, while hypertension control with labetalol was required at the emergence. Transesophageal echocardiography showed findings comparable to rest transthoracic echocardiography (Video S2a,b). Hemodynamic monitoring was performed with transesophageal echocardiography, monitoring central venous pressure (CVP), urine output, and trending lactic acid as marker of perfusion. No Swan-Ganz catheter was placed intraoperatively. The patient received a total of 1.8 L of crystalloids, 2 L of albumin, 6 units of packed red blood cells, 2 units of fresh frozen plasma, and 2 L of processed blood from cell saver. Blood loss was estimated to be of 3 L, and urine output was 1 L after 20 mg of furosemide. The patient was extubated in the operating room and transferred to the surgical ICU. During the postoperative period, volume status was monitored with CVP, non-invasive blood pressure, and trend of lactic acid. He did not require any diuretics while urine output was adequate, and he was started on nifedipine for hypertension, he required insulin to control hyperglycemia, and he complained of dyspnea on exertion, which was primarily attributed to acute postoperative anemia. The immunosuppressant medications consisted of induction with steroids, which were tapered until interruption, and maintenance with tacrolimus and mycophenolate mofetil. The patient was transferred to the floor on postoperative day (POD) 2 and discharged home on POD 6. However, on POD 8, he was readmitted for self-limited episodes of confusion, dyspnea, and leg swelling. An electrocardiogram showed T wave inversion in the anterior leads, Troponin I was 0.13 ng/mL (versus 0.04 on POD 5, upper limit of normal 0.08 ng/mL), and an arterial blood gas analysis showed hypoxemia and hypocapnia on room air. An echocardiogram revealed flattened interventricular septum as per right ventricle overload, severe dilation of right ventricle along with severely reduced systolic function, normal left and right atrial size, and an estimated pulmonary artery systolic pressure (PASP) of 70–75 mmHg (Video S3, Figure 3).

Deep vein thrombosis, pulmonary embolism, and acute coronary syndrome were ruled out with appropriate testing. The right heart catheterization was consistent with severe PAH (PH WHO Group 1, mean pulmonary artery (PA) pressure of 64 mmHg, wedge pressure of 16 mmHg, and pulmonary vascular resistance (PVR) of 579 dynes-s/cm^5^). There was no response to the nitric oxide vasodilation test. Considering that the patient had symptoms at rest and exhibited signs and symptoms of right heart failure (Class IV of the WHO functional classification), he was started on sildenafil (20 mg three times daily), a phosphodiesterase-5 inhibitor and continuous intravenous epoprostenol, a prostanoid, was titrated to 16 ng/kg/min. As there was no response to the nitric oxide vasodilation test, calcium channel blockers were not considered to be a therapeutic option. Other pharmaceutical options could have included endothelin receptor antagonists and guanylate cyclase stimulants. A follow-up echocardiogram 4 days after starting the PH treatment showed a slight improvement in the right ventricle systolic function and minor decrease in the right ventricle size. The patient was discharged after a week with no chest pain and with improved shortness of breath.

**Figure 4:**
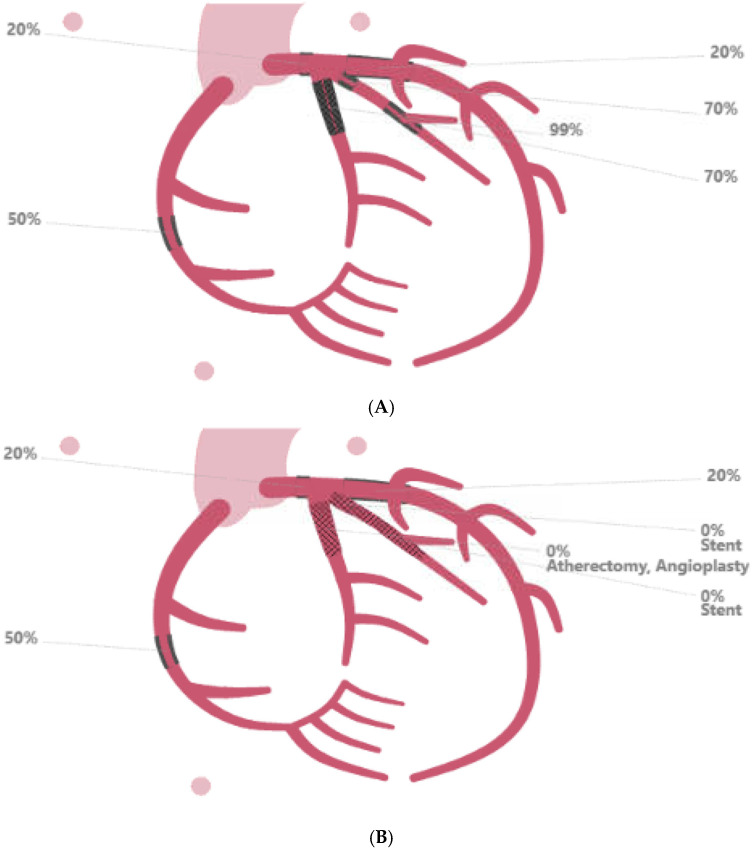
(**A**) diagnostic coronary angiography performed after liver transplant showed the worsening of coronary artery disease. Notably, in-stent restenosis of the left circumflex coronary artery (LCx) stent was observed; panel (**B**) shows the results of a complex percutaneous coronary intervention resulting in atherectomy and angioplasty of the LCx stent and the placement of a stent in the ramus intermedius.

Approximately 2.5 months later, the patient presented to the emergency department with worsening dyspnea and chest pain. An echocardiogram revealed new mild-to-moderate inferolateral wall hypokinesis with left ventricle ejection fraction of 60%, while EKG and troponin were unremarkable. The right ventricle was mildly dilated with normal systolic function. (Video S4). Coronary angiography showed severe coronary artery disease (70% stenosis of LMA, 99% stenosis of ostial LCx, and 99% in-stent restenosis of the proximal LCx) (Figure 4).

Due to the extent of the disease, cardiothoracic surgery was consulted, but the patient was deemed at prohibitive risk for coronary artery bypass graft. As a result, a complex percutaneous coronary intervention (PCI) was performed, including PCI of the in-stent restenosis and a placement of a DES in the ramus intermedius. The DAPT was reinstated along with guideline-directed medical therapy.

A concomitant right heart catheterization showed a favorable response to the vasodilatory therapy with a mean PA pressure of 26 mmHg, wedge pressure of 13 mmHg, right atrial pressure of 6 mmHg, and PVR of 106 dynes-s/cm^5^. The PH therapy remained unchanged. The patient was eventually discharged home asymptomatic and a 3-month follow-up was instituted.

## 3. Discussion

This case describes a rare scenario of rapid-onset, severe PAH diagnosed in the early postoperative period following liver transplantation in a patient without documented preoperative PH. Although the temporal relationship is striking, the available data do not allow definitive confirmation that the condition was truly *de novo*, as subclinical or masked pulmonary vascular disease prior to transplantation cannot be fully excluded.

PH is a well-recognized complication in patients with end-stage liver disease, primarily in the form of POPH, a subtype of PAH, which accounts for 65% of PH cases in this population [1,2]. Several cases of PH in cirrhotic patients improve or resolve following the liver transplant. Approximately 44% of patients with POPH undergoing liver transplant after medical optimization are able to wean off medication postoperatively [6].

By contrast, much less is known about the development of *de novo* PAH after liver transplantation, with the literature mainly consisting of case reports, suggesting that the incidence of this condition is exceedingly rare [4,5]. The case described by Koch et al. shares similarities with ours, as the patient had no evidence of pulmonary hypertension (PH) prior to surgery and developed PH in the immediate postoperative period [5]. However, in their report, recurrent biliary cirrhosis of the transplanted liver was documented, whereas our patient maintained normal graft function. Notably, the case reported by Alharbi et al. describes a patient who developed PH several years after transplantation without any signs of graft dysfunction, underscoring that this condition may also manifest as a late-onset complication [4].

The patient had risk factors that may have contributed to the development of postoperative PAH, including obesity-related OSA and CAD [7]. The exclusion of pulmonary embolism and left heart disease as etiologies of PH confirmed a primary vascular abnormality. The pathogenesis of post-liver transplant PAH is unknown. However, it has been suggested that PAH may be masked before transplantation by the co-presence of the hepatopulmonary syndrome (HPS), which causes pulmonary vasodilation. Immediately after transplantation, the resolution of HPS could unmask PAH. Another potential explanation is that the increased pulmonary blood flow characteristic of HPS may lead to vascular remodeling, which could then unmask PAH once the HPS is resolved through liver transplantation [7]. Of note, in this patient, there was no overt diagnosis of HPS, except for the presence of late appearance of microbubbles in the left heart chamber during the saline contrast study performed during the preoperative echocardiogram.

Interestingly, the patient showed a favorable response to the pulmonary vasodilator therapy, including sildenafil and intravenous epoprostenol, which led to significant hemodynamic improvement. This suggests that aggressive PAH-targeted therapy can be beneficial in post-transplant patients with newly diagnosed PAH. Given the association between PH and cardiovascular disease, it is also notable that the patient developed severe in-stent restenosis and underwent a complex PCI post-transplant. The interplay between PH and CAD in this setting remains an area of interest, particularly in the context of endothelial dysfunction and increased vascular reactivity seen in both conditions [8].

## 4. Conclusions

In conclusion, this case underscores the importance of vigilant post-transplant monitoring for pulmonary vascular complications, even in patients without pre-existing PH. PAH should be considered as a differential diagnosis in post-liver transplant patients with cardiovascular and respiratory symptoms. At the same time, caution is warranted in attributing causality, as the distinction between a truly *de novo* disease and previously unrecognized or unmasked pulmonary vascular pathology remains challenging in the absence of pre-transplant invasive hemodynamic assessment. Further investigation is warranted to clarify: (1) the true incidence of PAH diagnosed after liver transplantation; (2) the presence of identifiable risk factors or biomarkers that may predict its development; (3) whether preventive or pre-emptive therapeutic strategies could be implemented before transplantation; (4) the optimal management approaches after transplant; and (5) whether this condition is reversible.

Take-home messages:

- PAH can emerge early after liver transplantation, even in the absence of documented pre-transplant pulmonary hypertension.
- New cardiopulmonary symptoms after transplant warrant prompt evaluation for pulmonary vascular disease.
- Rapid initiation of guideline-directed PAH therapy can result in significant clinical and hemodynamic improvement, and a multidisciplinary approach is essential.
- It is important to maintain a broad differential diagnosis in early post-transplant deterioration.

## Data Availability

The original contributions presented in this study are included in the article and Supplementary Material. Further inquiries can be directed to the corresponding author.

## Supplementary Materials

The following supporting information can be downloaded at: https://www.mdpi.com/article/10.3390/reports9010083/s1, Video S1a: Preoperative transthoracic echocardiogram: apical four chambers view. Video S1b: Preoperative transthoracic echocardiogram: parasternal short axis view. Video S2a: Intraoperative transesophageal echocardiogram: mid-esophageal four chambers view. Video S2b: Intraoperative transesophageal echocardiogram: right ventricle inflow outflow view. Video S3: Transthoracic echocardiogram: postoperative day 8, apical four chambers view. Video S4: Transthoracic echocardiogram: apical four chambers view. Presence of new wall motion abnormalities.

## Abbreviations

The following abbreviations are used in this manuscript:

CAD	coronary artery disease
DAPT	dual antiplatelet therapy
DES	drug-eluting stent
HPS	hepatopulmonary syndrome
LAD	left anterior descending
LCx	left circumflex coronary artery
LMA	left main artery
OSAS	obstructive sleep apnea syndrome
PA	pulmonary artery
PAH	pulmonary arterial hypertension
PASP	pulmonary artery systolic pressure
PCI	percutaneous coronary intervention
PH	pulmonary hypertension
POD	postoperative day
POPH	portopulmonary hypertension
PVR	pulmonary vascular resistance
RCA	right coronary artery
